# Normative Values on Stromal Curvature, Power and Corneal‐Stromal Curvature Ratios From a Hybrid AS‐OCT

**DOI:** 10.1111/ceo.14556

**Published:** 2025-05-22

**Authors:** Jascha A. Wendelstein, Arianna Grendele, Achim Langenbucher, Alice Galzignato, Catarina Praefke Coutinho, Seth Pantanelli, Kamran M. Riaz, Giacomo Savini

**Affiliations:** ^1^ University Eye Hospital, Ludwig‐Maximilians‐University Munich Germany; ^2^ Institut für Refraktive und Ophthalmo‐Chirurgie (IROC) Zurich Switzerland; ^3^ Institute of Experimental Ophthalmology Saarland University Homburg Germany; ^4^ Studio Oculistico d'Azeglio Bologna Italy; ^5^ Dipartimento di Farmacia e Biotecnologie University of Bologna Bologna Italy; ^6^ Department of Ophthalmology Penn State College of Medicine Hershey Pennsylvania USA; ^7^ Dean A. McGee Eye Institute/University of Oklahoma Oklahoma City Oklahoma USA; ^8^ IRCCS Bietti Foundation Rome Italy

**Keywords:** anterior curvature, corneal radius, curvature ratio, posterior curvature, stromal curvature

## Abstract

**Background:**

To establish normative values for stromal curvature and its relationships to anterior and posterior curvature.

**Methods:**

Retrospective observational study. Data were obtained from 75 eyes of cataract surgery patients using a high‐resolution anterior segment optical coherence tomograph (AS‐OCT; MS‐39, CSO). Data analysis included anterior, stromal, and posterior curvature across multiple zones (1.0–5.0 mm), and derived curvature ratios.

**Results:**

The anterior‐to‐stromal curvature ratio (ASR) remained stable (1.016–1.007), while the stromal‐to‐posterior (SPR) and anterior‐to‐posterior (APR) ratios exhibited trends of slight change (SPR: 1.149–1.167; APR: 1.167–1.175). The refractive power analysis revealed incremental effects across one, two, and three refractive surface models, emphasising the importance of integrating posterior and stromal curvature data into optical assessments.

**Conclusion:**

This study provides the first normative values for stromal curvature and its ratios with anterior and posterior curvature. These findings may have implications for corneal‐ and lens‐based surgeries and diagnostics.

## 1 Introduction

The accurate assessment of corneal power is a cornerstone in ophthalmology, with critical implications for procedures such as cataract surgery, refractive surgery, and the management of corneal pathologies. Historically, keratometers and biometers have relied on measurements of the anterior corneal surface, combined with empirical adjustments for posterior corneal curvature and corneal thickness, to estimate total corneal power. However, these approaches have limitations, particularly in eyes with iatrogenically altered, pathologically irregular, or variably ectatic corneas, or with a history of laser vision correction (LVC). With the advent of modern anterior segment optical coherence tomographers (AS‐OCT) and tomography devices, besides corneal front and back surface, it is now possible to measure the surface geometry of the stromal surface directly, offering new opportunities for corneal power calculation in LVC and intraocular lens (IOL) power calculations [1, 2, 3].

The role of the stromal layer in corneal modelling is derived from its relative stability compared to the epithelium, which can remodel in response to irregularities. In cases of keratoconus and post‐LVC, where anterior corneal curvature can be markedly altered, stromal curvature provides a potentially less variable and therefore potentially more reliable parameter for diagnostic and predictive modelling. Traditional anterior‐to‐posterior curvature ratio (APR) models, while useful, are limited by their reliance on fixed or population‐averaged ratios that do not adequately capture subtle or masked abnormalities. Stromal curvature and its ratios to anterior and posterior curvature offer the potential to enhance the detection of irregularities in such challenging cases.

Our previous study has demonstrated the impact of epithelial remodelling on corneal power calculations, showing minimal effects in normal corneas but significant deviations in ectatic and post‐refractive cases, with differences of up to 0.9 diopters in certain scenarios [4]. These findings underscore the importance of integrating stromal‐specific parameters into optical models. However, a gap remains in the availability of normative values for stromal curvature and curvature ratios, which are essential for advancing corneal modelling and improving clinical outcomes. Insights into normative stromal curvature and its relationship to anterior and posterior corneal curvature could improve the accuracy of IOL power calculations, enhance the detection of ectasia and LVC‐related irregularities, and inform the development of advanced surgical planning tools.

This study seeks to address this gap by establishing normative values for stromal curvature and its relationships to anterior and posterior curvature. Using AS‐OCT, we analyse anterior‐to‐stromal (ASR), stromal‐to‐posterior (SPR), and anterior‐to‐posterior (APR) corneal curvature ratios across multiple measurement zones. To our knowledge, these data provide the first comprehensive reference for stromal curvature and its ratios. We hypothesise that stromal curvature and its ratios to anterior and posterior curvature will provide stable, normative benchmarks for improving corneal power assessments.

## 2 Methods

### 2.1 Study Design

This retrospective study conformed to ethics codes based on the tenets of the Declaration of Helsinki. Prior ethics approval was obtained (Ärztekammer des Saarlandes, 157/21). We included the eyes of cataract surgery patients older than 18 years.

### 2.2 Parameters Used for Calculation

**FIGURE 1:**
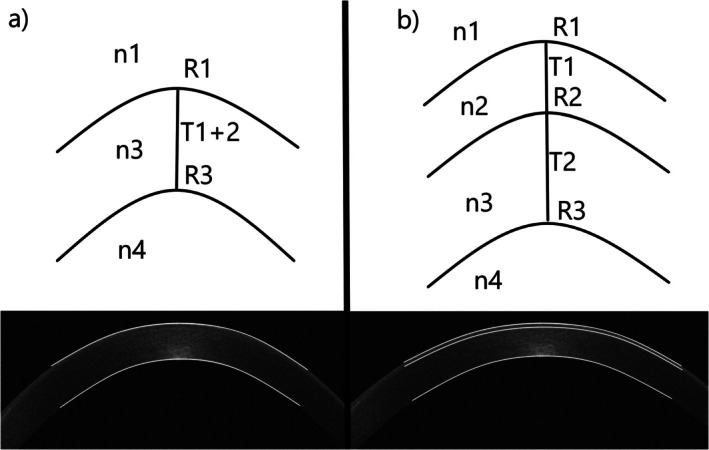
Schematic scheme of 2 (a) and 3 (b) refractive surface models. Anterior to posterior curvature ratio (APR) is displayed by surfaces R1/R3, and anterior to stromal curvature ratio (ASR) and stromal to posterior curvature ratio (SPR) are displayed by surfaces R1/R2 and R2/R3, respectively.

For the refractive indices, we use 1.0 for air and literature data for the epithelium, the stroma, and the aqueous humour [5, 6, 7, 8]. Parameters considered in calculations (Figure 1) are:

1. Refractive index of air (*n*_1_ = 1.0)
2. Refractive index of the epithelium (*n*_2_ = 1.40)
3. Refractive index of the stroma/cornea (*n*_3_ = 1.376)
4. Refractive index of the aqueous (*n*_4_ = 1.336)
5. Keratometric index referenced to front surface vertex plane (FV) power (*n*_*K*_ = 1.332)
6. Epithelial front radius of curvature (*R*_1_)
7. Stromal front radius of curvature (*R*_2_)
8. Corneal back surface radius of curvature (*R*_3_)
9. Central epithelial thickness (*T*_1_) measured from the anterior surface down to the anterior surface of the Bowman's layer
10. Central stromal thickness (*T*_2_) From the anterior Bowman's layer down to the posterior surface of the endothelial layer
11. Central total corneal thickness (*T*_1+2_)
12. Corneal power (*F*_C_)
13. Power of the epithelial front surface (*F*_1_)
14. Power of the stromal front surface (*F*_2_)
15. Power of the corneal back surface (*F*_3_)

Calculations of corneal power with 1, 2, and 3 refractive surfaces were performed as described in our previous publication [4].

### 2.3 Data Export

A unilateral dataset (*n* = 75) comprising clinical data from 75 consecutive patients at the IRCCS Bietti Foundation (Rome, Italy) was included in this retrospective study. Eyes were excluded if they had known anterior (e.g., anterior basement membrane dystrophy, keratoconus, pellucid) or posterior (Fuchs) corneal degenerations or dystrophies, a history of trauma or identifiable scars, or prior ocular surgery of any kind other than laser iridectomy/retinopexy. All data, including patient names and dates of birth, were anonymized at the source and stored in an Excel (Microsoft Corporation) file, which was subsequently transferred to the Department of Experimental Ophthalmology at Saarland University, Germany, for further analysis. Measurement data were obtained using an AS‐OCT (MS‐39, CSO, Italy) and exported with the standard user software. The dataset included corneal curvature, power, and surface height measurements (epithelium, stroma, and endothelium) organised in a cylindrical coordinate system with 256 equidistant semimeridians and 30 radial distances, ranging from 0.2 to 6 mm from the center in 0.2 mm increments.

For spherical fit analysis, all 256 × 30 data points within the 12 mm zone were evaluated. A floating best‐fit sphere was fitted to the epithelium, stroma, and endothelium within central zones of 1.0, 2.0, 3.0, 4.0, and 5.0 mm diameters by minimising the root‐mean‐square fit error. From these fits, the mean radii of curvature and sphere centres were determined. The extracted radii and apices were then used for paraxial calculations.

### 2.4 Statistical Analysis

In our study, we selected a sample size of 75 patients to examine the ASR within the 3.0 to 5.0 mm zones, areas commonly assessed by standard ophthalmic devices such as biometers, keratometers, and tomographers. To determine if this sample size is sufficient, a sample size calculation was performed. The sample size was determined based on the standard deviations observed in these zones (0.010 at 3.0 mm, 0.008 at 4.0 mm, and 0.007 at 5.0 mm), aiming for a 95% confidence interval with a margin of error of ±0.005. The calculated required sample sizes were approximately 16, 10, and 8 for the 3.0, 4.0, and 5.0 mm zones, respectively. Therefore, our chosen sample size of 75 provides a robust margin, ensuring precise and reliable estimation of the ASR across these critical measurement zones.

Statistical analysis was performed with IBM SPSS version 29.0.0.0. Only unilateral data was considered. Data are listed exploratively in terms of the arithmetic mean, standard deviation (SD), median, and the lower and upper boundaries of the 95% confidence interval (2.5% and 97.5% quantiles).

The normality of data distribution was assessed using the Shapiro–Wilk test. To evaluate differences in parameters across various central measurement zones (1, 2, 3, 4, and 5 mm), a repeated measures analysis of variance (rmANOVA) was performed for normally distributed data. If the assumption of normality was violated, the non‐parametric Friedman test was used instead. Post hoc pairwise comparisons between specific zones were conducted using paired *t*‐tests for normally distributed differences, or Wilcoxon signed‐rank tests otherwise. To compare two corneal parameters derived from the same measurement zone, paired‐sample statistical tests were applied analogously: paired *t*‐tests for normally distributed differences, or Wilcoxon signed‐rank tests in the absence of normality. To correct for multiple comparisons, the Benjamini–Hochberg procedure was applied to control the false discovery rate at a threshold of 5%.

## 3 Results

The analysis dataset included 75 eyes from 75 patients from the IRCCS Bietti Foundation (Rome, Italy). The female/male ratio was 64% to 36%. The mean age of the 75 patients was 62.25 ± 8.68 years (Range: 25–88 years). Fifty‐two % left eyes and 48% right eyes (39/36) were included (Table S1).

### 3.1 Corneal Curvature Analysis

The mean anterior corneal radius of curvature increased progressively with expanding measurement zones, while the corresponding standard deviations decreased, indicating reduced variability in peripheral measurements (Table 1). Post hoc analysis revealed a zone‐dependent curvature pattern, with 9 out of 10 pairwise comparisons reaching statistical significance, except between the 4 and 5 mm zones.

**TABLE 1:** Anterior, stromal and posterior radius of corneal curvature in all eyes (mm).

	Mean	SD	Median	IQR	97.5% quantile	2.5% quantile
Anterior						
1.0 mm	7.690	0.295	7.669	0.234	8.293	7.255
2.0 mm	7.700	0.286	7.680	0.226	8.265	7.272
3.0 mm	7.708	0.281	7.685	0.223	8.263	7.279
4.0 mm	7.724	0.274	7.698	0.222	8.265	7.293
5.0 mm	7.744	0.266	7.717	0.224	8.276	7.306
Stromal						
1.0 mm	7.572	0.318	7.576	0.281	8.059	6.976
2.0 mm	7.639	0.288	7.625	0.337	8.131	7.180
3.0 mm	7.653	0.283	7.635	0.294	8.198	7.192
4.0 mm	7.671	0.282	7.660	0.247	8.239	7.213
5.0 mm	7.693	0.278	7.688	0.265	8.260	7.241
Posterior						
1.0 mm	6.592	0.228	6.612	0.268	6.976	6.110
2.0 mm	6.600	0.212	6.608	0.270	6.978	6.167
3.0 mm	6.589	0.205	6.592	0.225	6.993	6.178
4.0 mm	6.583	0.201	6.595	0.218	6.998	6.187
5.0 mm	6.591	0.200	6.597	0.222	6.997	6.179

At each corresponding radial distance, the anterior stroma exhibited a smaller radius of curvature compared to the anterior epithelium, signifying a consistently steeper profile. Moreover, the anterior stroma demonstrated a more pronounced increase in radius of curvature from the center to the periphery, suggesting a more prolate configuration. In contrast, the anterior epithelium maintained a relatively uniform curvature, indicative of a less prolate or more spherical shape (Table 1). Similar to the anterior surface findings, 8 out of 10 pairwise comparisons for the stroma were significant, with non‐significant differences observed between the 2–3 and 4–5 mm zones.

Regarding the posterior corneal surface, curvature measurements remained relatively consistent across all zones (Table 1). Only 2 out of 10 pairwise comparisons (specifically between the 2–3 and 2–4 mm zones) reached statistical significance, suggesting minimal curvature variation across these regions.

### 3.2 Surface Power Analysis

The mean anterior corneal power exhibited a slight decrease from the central to peripheral zones, suggesting a gradual flattening of the anterior corneal surface towards the periphery (Table 2). Correspondingly, the standard deviation (SD) decreased indicating reduced variability in peripheral measurements.

**TABLE 2:** Front, stromal and back surface power in all eyes (in diopters).

	Mean	SD	Median	IQR	97.5% quantile	2.5% quantile
Anterior						
1.0 mm	52.086	1.893	52.156	1.588	55.133	48.248
2.0 mm	52.015	1.828	52.081	1.536	55.008	48.408
3.0 mm	51.956	1.799	52.047	1.510	54.954	48.416
4.0 mm	51.850	1.754	51.962	1.496	54.851	48.405
5.0 mm	51.713	1.700	51.835	1.502	54.750	48.334
Stromal						
1.0 mm	−3.175	0.130	−3.168	0.118	−2.978	−3.441
2.0 mm	−3.146	0.115	−3.148	0.139	−2.952	−3.343
3.0 mm	−3.140	0.112	−3.143	0.121	−2.928	−3.337
4.0 mm	−3.133	0.111	−3.133	0.101	−2.913	−3.327
5.0 mm	−3.123	0.109	−3.122	0.108	−2.906	−3.315
Posterior						
1.0 mm	−6.076	0.211	−6.049	0.247	−5.734	−6.547
2.0 mm	−6.066	0.195	−6.053	0.248	−5.732	−6.487
3.0 mm	−6.076	0.189	−6.068	0.207	−5.720	−6.475
4.0 mm	−6.082	0.185	−6.066	0.201	−5.716	−6.465
5.0 mm	−6.074	0.184	−6.064	0.205	−5.717	−6.474

The stromal surface demonstrated a progressive increase in mean refractive power (more negative values), reflecting a subtle steepening towards the periphery. The SD decreased across these zones, indicating enhanced measurement consistency in peripheral regions (Table 2). Finally, the posterior corneal surface maintained a relatively uniform mean power across all measured zones (Table 2). The SD remained stable, indicating minimal variability and a stable posterior curvature profile.

**FIGURE 2:**
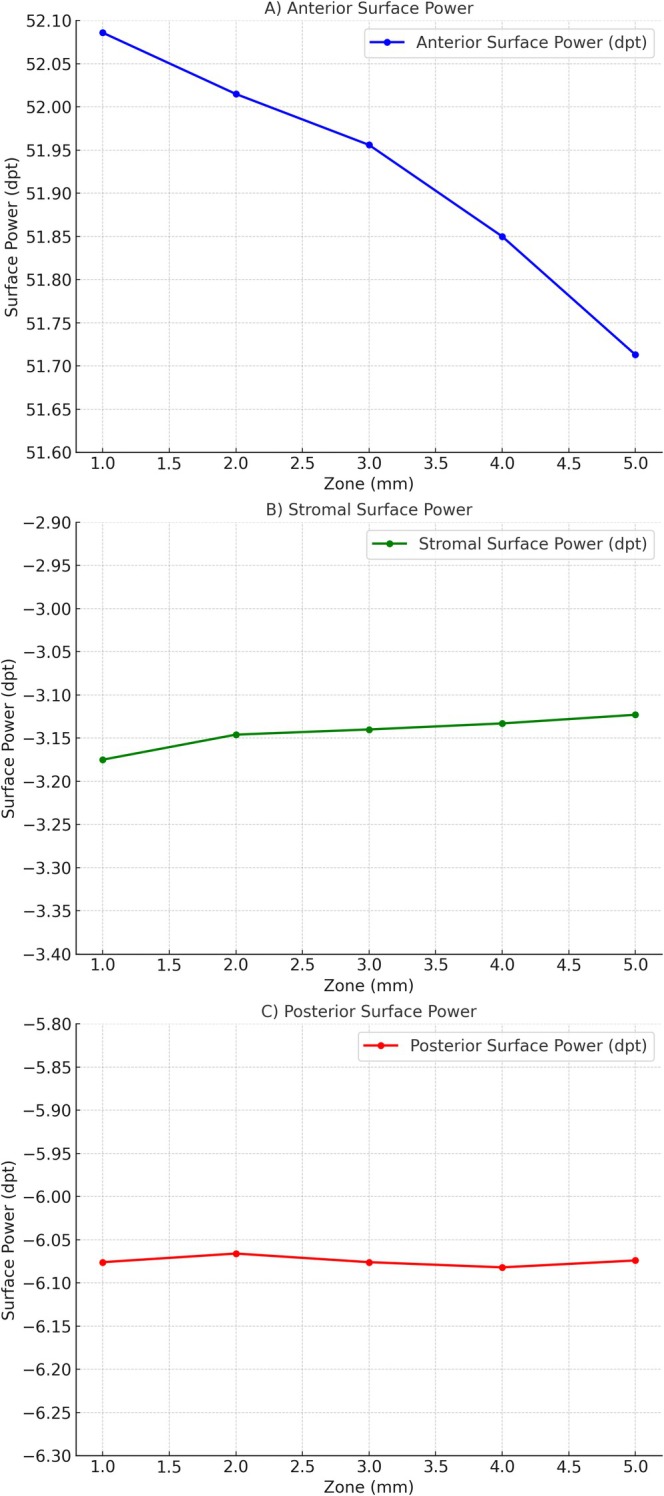
This figure presents the surface power as a function of radial zone diameter across the anterior, stromal, and posterior corneal layers over measurement zones ranging from 1.0 to 5.0 mm.

These trends indicate that the stromal and posterior surfaces contribute less but still considerably to the overall refractive power compared to the anterior surface (Figure 2).

### 3.3 Curvature Ratios

**FIGURE 3:**
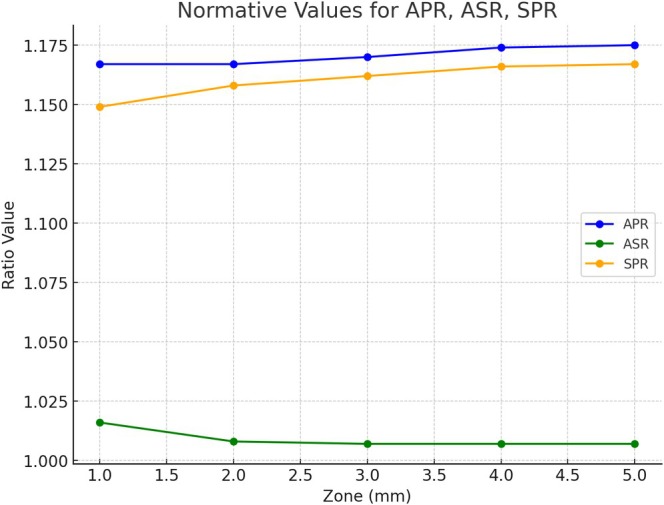
This figure illustrates the radius of curvature ratios—Anterior‐to‐Stromal Ratio (ASR), Stromal‐to‐Posterior Ratio (SPR), and Anterior‐to‐Posterior Ratio (APR)—across measurement zones from 1.0 to 5.0 mm.

The ASR reflects the cornea's natural prolate shape, where the central curvature is steeper than the peripheral curvature and decreases minimally towards the periphery, indicating that the anterior surface becomes relatively flatter compared to the stromal surface towards the periphery. The decrease was small, hence only the 1 mm zone showed significant differences to all other zones (Table 3 and Figure 3), while the other zones seemed to be rather stable. The SD also decreased, suggesting increased uniformity in this ratio in the peripheral zones.

**TABLE 3:** This table presents three key ratios that describe the relationships between the radii of curvature of different corneal surfaces: ASR (Anterior‐to‐Stromal Radius Ratio), SPR (Stromal‐to‐Posterior Radius Ratio), APR (Anterior‐to‐Posterior Radius Ratio) in all eyes.

	Mean	SD	Median	IQR	97.5% quantile	2.5% quantile
Anterior‐stromal ratio						
1.0 mm	1.016	0.024	1.015	0.020	1.070	0.976
2.0 mm	1.008	0.013	1.008	0.011	1.037	0.979
3.0 mm	1.007	0.010	1.008	0.010	1.026	0.982
4.0 mm	1.007	0.008	1.007	0.009	1.022	0.989
5.0 mm	1.007	0.007	1.007	0.007	1.020	0.990
Stromal‐posterior ratio						
1.0 mm	1.149	0.040	1.144	0.038	1.209	1.081
2.0 mm	1.158	0.034	1.155	0.027	1.221	1.116
3.0 mm	1.162	0.031	1.159	0.026	1.213	1.122
4.0 mm	1.166	0.029	1.163	0.028	1.212	1.130
5.0 mm	1.167	0.029	1.166	0.026	1.211	1.134
Anterior–posterior ratio						
1.0 mm	1.167	0.040	1.161	0.041	1.235	1.106
2.0 mm	1.167	0.035	1.164	0.031	1.220	1.120
3.0 mm	1.170	0.033	1.165	0.029	1.219	1.128
4.0 mm	1.174	0.031	1.168	0.026	1.216	1.135
5.0 mm	1.175	0.029	1.170	0.027	1.216	1.138

The SPR increased towards the periphery and suggests a differential flattening pattern between these layers, with a corresponding decrease in SD. This indicates that the stromal surface becomes relatively flatter compared to the posterior surface towards the periphery. It showed the largest differences between central and peripheral zones among all three ratios (Table 3 and Figure 3). All paired zones showed significant differences except the 1 –2 mm and 3 –5 mm zones.

APR showed slightly higher values than SPR but a more moderate peripheral decrease, with significant differences in all but three comparisons (1–2, 1–3, and 4–5 mm). In each zone, all ratios were systematically different (Table 3 and Figure 3).

### 3.4 Central Corneal Thickness (CCT), Central Stromal Thickness (CST) and Central Epithelial Thickness (CET)

The mean CCT and CST were remarkably consistent across zones. Mean CCT was approximately 0.541 mm, while CST averaged 0.487 mm. The consistency across zones highlights the uniformity of corneal and stromal thickness across the measurement zones (Table 4). Epithelial thickness (CET) remained stable at a mean of 0.053 mm across all zones, with minimal deviation observed. The narrow interquartile ranges and low SD values indicate limited variation in epithelial thickness among the studied cohort (Table 4).

**TABLE 4:** Central corneal (C), central stromal (S), and central epithelial (E) thickness in all eyes (in mm).

	Corneal layer (s)	Mean	SD	Median	IQR	97.5% quantile	2.5% quantile
1.0 mm	C	0.541	0.032	0.541	0.047	0.596	0.487
	S	0.488	0.032	0.487	0.047	0.543	0.429
	E	0.053	0.004	0.053	0.005	0.061	0.046
2.0 mm	C	0.541	0.032	0.542	0.045	0.595	0.487
	S	0.488	0.032	0.490	0.043	0.542	0.429
	E	0.053	0.004	0.053	0.005	0.061	0.046
3.0 mm	C	0.541	0.032	0.542	0.044	0.595	0.487
	S	0.487	0.032	0.489	0.042	0.543	0.429
	E	0.053	0.004	0.053	0.005	0.061	0.046
4.0 mm	C	0.540	0.032	0.541	0.044	0.595	0.487
	S	0.487	0.032	0.489	0.042	0.543	0.429
	E	0.053	0.004	0.053	0.006	0.061	0.046
5.0 mm	C	0.540	0.032	0.541	0.045	0.596	0.486
	S	0.487	0.032	0.488	0.043	0.543	0.429
	E	0.053	0.004	0.053	0.006	0.061	0.046

### 3.5 Corneal Power Analysis

**FIGURE 4:**
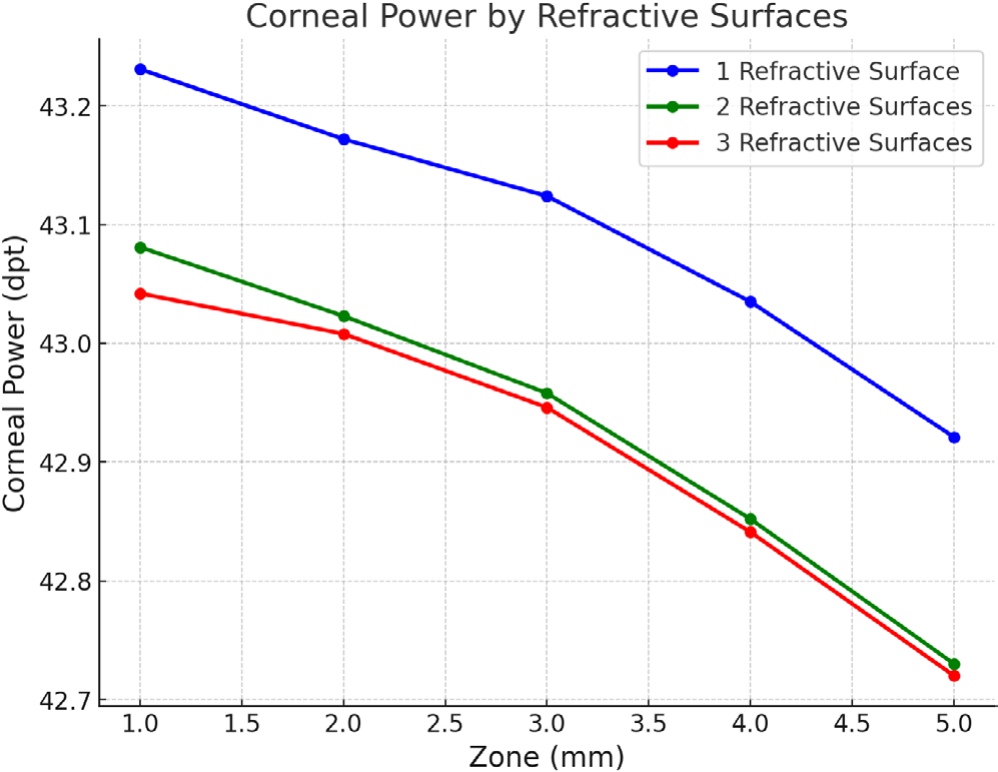
This figure depicts the corneal power across one (anterior surface, blue line), two (anterior and posterior surfaces, green line), and three (anterior, stromal, and posterior surfaces, red line) refractive surfaces models over measurement zones ranging from 1.0 to 5.0 mm. The one‐surface model demonstrates a gradual decline in mean power, starting at 43.231 D at 1.0 mm and decreasing to 42.921 D at 5.0 mm. The two‐ and three‐surface models follow similar trends with slightly lower power values, highlighting the incremental contributions of additional refractive surfaces.

The analysis of corneal power (referenced to the front surface plane) across 1, 2, and 3 refractive surfaces revealed slight variations. The 1‐refractive surface model showed a gradual decline in mean power from 43.231 D at 1.0 mm to 42.921 D at 5.0 mm. Similar trends were observed in the 2‐ and 3‐surface models, though with slightly lower values, indicating the incremental effect of additional refractive surfaces (Table 5 and Figure 4). Comparisons of power values computed using 1‐, 2‐, and 3‐surface models showed significant differences within each zone. Finally, corneal power calculations using the three‐refractive‐surface model revealed significant differences between all measurement zones, except between 1–2 and 4–5 mm.

**TABLE 5:** Corneal power in all eyes (in diopters). Presented are 1 refractive surface (anterior surface), 2 refractive surfaces (anterior and posterior surface) and 3 refractive surfaces (anterior, stromal, posterior surface) models. All three models are referenced to the front vertex plane.

	Refractive surfaces	Mean	SD	Median	IQR	97.5% quantile	2.5% quantile
1.0 mm	1	43.231	1.571	43.289	1.318	45.761	40.046
	2	43.081	1.677	43.119	1.434	45.671	39.949
	3	43.042	1.689	43.075	1.531	45.624	39.875
2.0 mm	1	43.172	1.517	43.227	1.275	45.656	40.179
	2	43.023	1.607	43.125	1.446	45.550	40.042
	3	43.008	1.612	43.115	1.502	45.519	40.002
3.0 mm	1	43.124	1.493	43.199	1.253	45.612	40.185
	2	42.958	1.577	43.085	1.418	45.488	40.048
	3	42.946	1.578	43.081	1.487	45.461	40.034
4.0 mm	1	43.035	1.456	43.128	1.242	45.526	40.176
	2	42.852	1.530	43.012	1.405	45.385	40.030
	3	42.841	1.529	42.994	1.464	45.365	40.030
5.0 mm	1	42.921	1.411	43.023	1.247	45.443	40.117
	2	42.730	1.477	42.931	1.291	45.303	39.962
	3	42.720	1.474	42.916	1.307	45.286	39.964

## 4 Discussion

This study represents a significant step forward in understanding the structural and optical characteristics of the cornea through AS‐OCT. By establishing normative values for stromal curvature and its ratios to anterior and posterior curvature, we provide a foundational reference for clinical and research applications. To our knowledge, this is the first study to report normative stromal curvature power and radius across multiple measurement zones, as well as APR in various zones rather than a single zone. Additionally, it introduces and establishes normative data for two novel curvature ratios: the ASR and the SPR, both analysed across different measurement zones.

Historically, corneal power calculations relied heavily on empirical adjustments to anterior curvature measurements, often neglecting the contributions of posterior and stromal layers. Our findings highlight the limitations of single‐surface corneal models derived from traditional keratometer indices, based on the Gullstrand model eye, which tend to overestimate corneal power. Incorporating stromal and posterior curvature data has the potential to enhance the accuracy of optical models, particularly for applications such as IOL power calculations. The ASR, SPR, and APR demonstrated consistency across measurement zones, highlighting the structural proportionality of the corneal layers. These ratios could serve as reliable parameters in corneal modelling and could be particularly useful for preoperative screening of LVC, keratoconus, and other irregular corneas in cataract surgery [9].

The study device and our surface fit model demonstrated an APR of 1.167 in the 2.0 mm zone, 1.170 in the 3.0 mm zone, and 1.174 in the 4.0 mm zone. Our values are notably higher than the APR reported for the IOLMaster 700 (Carl Zeiss Meditec AG, Jena, Germany), which is approximately 1.12 for a measurement zone of around 3.0 to 3.5 mm [10, 11, 12]. However, our values are slightly lower than those reported for several other devices. For instance, the Anterion (Heidelberg Engineering, Heidelberg, Germany) and Casia SS‐1000 (Tomey) exhibited an APR of approximately 1.19 for measurement zones of 3.0 and 3.0 mm, respectively [12, 13]. The Sirius (CSO SRL, Italy) showed an APR of around 1.20 for a measurement zone of 4.0 to 5.0 mm [14]. Finally, the Pentacam (Oculus, Wetzlar, Germany) reported an APR ranging from 1.21 to 1.22 for a measurement zone of 3.0 to 4.0 mm [15, 16, 17, 18]. The standard deviations for ASR, SPR, and APR were 0.013, 0.034, and 0.035, respectively, in the 2.0 mm zone; 0.010, 0.031, and 0.033 in the 3.0 mm zone; and 0.008, 0.029, and 0.031 in the 4.0 mm zone. Our findings are not only different from these various devices, but further underscore the importance of understanding and appreciating that corneal power measurements from one device are not interchangeable with another device. Ideally, these devices would offer a display mode that presents curvature and curvature ratios across multiple measurement zones. While this feature might seem unnecessary when most IOL formulas rely solely on anterior curvature, it becomes crucial when employing thick cornea models or ray tracing techniques. In such cases, discrepancies in the APR across devices can lead to significant errors in IOL power calculations if incorrect values are input into the formulas. Moreover, formulas designed to adjust calculations for conditions like keratoconus or post‐LVC eyes may also be compromised. These formulas often use posterior curvature to estimate changes resulting from ectasia or ablation, integrating this information to predict postoperative refraction. Inaccurate APR measurements can thus adversely affect these predictions. Finally, any ray tracing calculation—whether for IOL power determination or LVC ablation profile design—can be impacted by systematic differences in anterior and/or posterior curvature measurements. This underscores the importance of standardised measurements and the need for devices to provide comprehensive curvature data across various zones to ensure accurate refractive outcomes. Having established normative values for a device, the next intriguing question is how much the anterior‐to‐stroma ratio varies in non‐normal eyes, such as post‐LVC or keratoconus cases.

Similar to Zhou et al., we observed a steeper curvature of the anterior stromal surface compared to the epithelialised anterior corneal surface, which indicates that the corneal epithelium contributes to a flattening effect on the anterior corneal surface [19]. This flattening effect reduces the cornea's natural prolateness by smoothing out curvature variations. In the study by Zhou et al., intraoperative swept‐source OCT measurements taken immediately after mechanical epithelial removal revealed a steeper stromal BFS radius by 1.28 diopters, along with increased astigmatism and higher‐order aberrations. The authors reported minimal influence of the observed stromal edema, with an average increase in stromal thickness of 19.47 μm. In contrast, our investigations did not observe a significant contribution of 1.28 D to corneal power in normal corneas. Our comparative analysis between two‐surface and three‐surface corneal models demonstrated differences of less than 0.1 D, with an ASR ratio of approximately 1.01. However, in specific conditions such as keratoconus or post‐laser vision correction, we identified more pronounced differences due to altered stromal curvature [4, 20].

The discrepancy between our findings and those of Zhou et al. may be influenced by several factors. Firstly, the stromal edema observed post‐epithelial removal in their study could have affected the measurements more than anticipated. Secondly, measurements taken immediately after deepithelialization might be more susceptible to variability. Lastly, differences in measurement devices could play a role; our study utilised the MS‐39 device, whereas Zhou et al. employed the Casia SS‐1000. We cannot conclusively comment on the epithelium's capacity to mask underlying stromal irregularities. Further research is necessary to elucidate the extent and variability of the epithelial masking effect on higher order aberrations across different corneal conditions.

The use of one, two, or three refractive surface models allowed for a nuanced assessment of corneal power, revealing how incremental contributions from each layer and various measurement zones affect overall optical outcomes (Figures 3 and 4). Anecdotally, the comparison between two‐surface and three‐surface models aligns with our clinical observations, confirming that back‐calculated corneal powers are consistently slightly lower than those estimated by a two‐surface model. This approach sets the stage for more refined IOL power calculation algorithms, particularly for challenging cases such as post‐refractive surgery eyes or corneal pathologies. It is of notice that the normative values for corneal power with a one‐, two‐, and three‐refractive surface models established in this study were referenced to the front vertex plane of the cornea. Therefore, we used a keratometer index of 1.332 for the single refractive surface model (which is only valid for the Gullstrand model eye). Instead, if we reference the back vertex plane by using the popular 1.3375 index value, the differences in power increase by more than 0.5 dpt. Of notice, if we switch to the back vertex plane, BVD, ACD, and AL will need to be calculated from the posterior cornea for paraxial calculations. A keratometer index is only related to the 1‐surface model, whereas in the 2‐surfaces model we use the refractive indices for air/cornea/aqueous and in the 3‐surfaces model we use the refractive indices for air/epithelium/stroma/aqueous.

Clinically, the normative values and curvature ratios reported in this study may have future implications. They could inform the development of advanced surgical planning tools, improve the accuracy of refractive surgery ablation profiles, and enhance the predictability of outcomes in cataract and refractive surgery. They could also be used to detect/monitor disease states that alter the APR/SPR/ASR, such as keratoconus, Fuchs disease, and others. Additionally, the consistent stromal thickness and curvature measurements across zones (measured as distances between apices) emphasise the uniformity of the corneal stroma, providing a reliable baseline for comparative studies. Notably, consistent stromal thickness—whether measured axially or perpendicularly—does not imply that the anterior surface is a scaled version of the posterior surface, as a shift in the apices can still occur.

However, this study has limitations. The dataset, while robust, was derived from a single imaging device and patient cohort with similar ethnic variation, which may limit the generalisability of the findings. Future studies should aim to validate these results across a more diverse population and include comparisons with other imaging modalities. Furthermore, we employed a floating best‐fit sphere for surface fitting. While this approach appears adequate for normal corneas, it may not be optimal for irregular corneas. Investigating alternative surface fit models, such as conic fits, could enhance our understanding of corneal geometry and its optical properties, particularly in cases with irregularities. Finally, corneal power in our study is dependent on established refractive indices (e.g., epithelium *n*_2_ = 1.40; stroma/cornea *n*_3_ = 1.376) and may change with updates on refractive indices.

Overall, this is the first study to explore normative values of the stromal curvature with modern AS‐OCT data. The data establish normative values for anterior, stromal, and posterior corneal curvatures and power across measurement zones. The curvature ratios provide insights into the structural proportionality of the corneal layers, while the consistent thickness measurements across zones underscore the uniformity of corneal anatomy. Having established normative values, the next intriguing question is how much the anterior‐to‐stroma ratio varies in non‐normal eyes, such as post‐LVC or keratoconus cases.

## Conflicts of Interest

Dr. Wendelstein reports research support from Carl Zeiss Meditec A.G. He reports personal fees from Alcon Surgical, Bausch and Lomb, Carl Zeiss Meditec AG, Heidelberg Engineering, Rayner Surgical, and Johnson & Johnson Vision outside of the submitted work. He was supported by an “ESCRS Peter Barry Fellowship Grant”. Dr. Riaz reports consulting fees from ImmunoGen and Ambrx Pharmaceuticals and personal fees from CorneaGen outside of the submitted work. Dr. Pantanelli receives research support from Alcon, Bausch and Lomb, and Carl Zeiss Meditec outside of the submitted work. He is also a consultant for Bausch and Lomb and Carl Zeiss Meditec AG, outside of the submitted work. Dr. Langenbucher reports personal fees from Hoya Surgical and Johnson & Johnson Vision outside the submitted work. Dr. Savini has received personal fees from Alcon, Johnson & Johnson, SIFI, Thea and Zeiss. Eng. Coutinho has received personal fees from Zeiss. The other authors declare no conflicts of interest.

## Supporting information


**Table S1.** Cohort description.

## Data Availability

The data that support the findings of this study are available from the corresponding author upon reasonable request.
