# Supplementary material for: Normative Values on Stromal Curvature, Power and Corneal‐Stromal Curvature Ratios From a Hybrid AS‐OCT
Source: Clin Exp Ophthalmol. 2025 May 22;53(7):746–55. doi: 10.1111/ceo.14556 (PMC12516649; doi:10.1111/ceo.14556)
Supplement: Supplementary file 1 — Table S1. Cohort description. [file CEO-53-746-s001.docx]

| N | 75 |
| --- | --- |
| Female/Male | 64% / 36% |
| OD/OS | 48% / 52% |
| Age | 62.25±8.68 |
| Supplementary table 1: Cohort description | |
